# Serial assessment of the physiological status of leatherback turtles (*Dermochelys coriacea*) during direct capture events in the northwestern Atlantic Ocean: comparison of post-capture and pre-release data

**DOI:** 10.1093/conphys/cou048

**Published:** 2014-10-30

**Authors:** Charles J. Innis, Constance Merigo, Julie M. Cavin, Kathleen Hunt, Kara L. Dodge, Molly Lutcavage

**Affiliations:** 1 Animal Health Department, New England Aquarium, Boston, MA 02110, USA; 2 Rescue and Rehabilitation Department, New England Aquarium, Boston, MA 02110, USA; 3 John H. Prescott Marine Laboratory, Research Department, New England Aquarium, Boston, MA 02110, USA; 4 Department of Biological Sciences, University of New Hampshire, Durham, NH 03824, USA; 5 Large Pelagics Research Center, University of Massachusetts Amherst, Gloucester, MA 01930, USA

**Keywords:** Capture, leatherback turtle, physiology

## Abstract

The physiological status of seven leatherback turtles (*Dermochelys coriacea*) was assessed at two time points during ecological research capture events in the northwestern Atlantic Ocean. Data were collected as soon as possible after securing each turtle onboard the capture vessel and again immediately prior to release. Measured parameters included sea surface temperature, body temperature, morphometric data, sex, heart rate, respiratory rate and various haematological and blood biochemical variables. Results indicated generally stable physiological status in comparison to previously published studies of this species. However, blood pH and blood potassium concentrations increased significantly between the two time points (*P* = 0.0018 and *P* = 0.0452, respectively). Turtles were affected by a mild initial acidosis (mean [SD] temperature-corrected pH = 7.29 [0.07]), and blood pH increased prior to release (mean [SD] = 7.39 [0.07]). Initial blood potassium concentrations were considered normal (mean [SD] = 4.2 [0.9] mmol/l), but turtles experienced a mild to moderate increase in blood potassium concentrations during the event (mean [SD] pre-release potassium = 5.9 [1.7] mmol/l, maximum = 8.5 mmol/l). While these data support the general safety of direct capture for study of this species, the observed changes in blood potassium concentrations are of potential concern due to possible adverse effects of hyperkalaemia on cardiac function. The results of this study highlight the importance of physiological monitoring during scientific capture events. The results are also likely to be relevant to unintentional leatherback capture events (e.g. fisheries interactions), when interactions may be more prolonged or extreme.

## Abstract (precis)

A variety of health parameters were evaluated serially for endangered leatherback turtles as they were captured in the northwestern Atlantic Ocean as part of an ecology study. Results indicated that turtles were healthy and capture events were smoothly conducted, but changes in blood pH and potassium concentrations were detected.

## Introduction

The leatherback turtle (*Dermochelys coriacea*) is the largest living species of turtle. It is listed as ‘vulnerable’ by the World Conservation Union (Wallace *et al.*, 2013) and as ‘endangered’ under the United States Endangered Species Act (USFW, 2014). Global threats to the species include the following: harvesting of eggs and adults for human consumption (Eckert and Sarti, 1997; Spotila *et al.*, 2000); loss, degradation and artificial lighting of nesting habitat (Lutcavage *et al.*, 1997; Deem *et al.*, 2007); ingestion of and entanglement in marine debris (Balazs, 1985; Mrosovsky *et al.*, 2009); effects of climate change on ocean productivity (Wallace *et al.*, 2006; Saba *et al.*, 2008); and mortality from fishery interactions (National Research Council, 1990; Lewison *et al.*, 2004a). Leatherback turtles migrate great distances between temperate foraging and tropical nesting sites and are therefore at risk from both pelagic fisheries (e.g. longline fisheries; Witzell, 1996; Lewison *et al.*, 2004b; López-Mendilaharsu *et al.*, 2009) and inshore fisheries (e.g. fixed-gear fisheries; Lutcavage and Musick, 1985; Godley *et al.*, 1998; Dwyer-Dodge *et al.*, 2002; James *et al.*, 2005; Alfaro-Shigueto *et al.*, 2007; López-Mendilaharsu *et al.*, 2009).

Studies employing direct capture of leatherback turtles at sea have provided substantial insight into the foraging ecology, habitat selection, migration and health of leatherback turtles of different sexes and life stages (James and Mrosovsky, 2004; James *et al.*, 2005, 2007; Benson *et al.*, 2006; Doyle *et al.*, 2008; Innis *et al.*, 2010; Benson *et al.*, 2011; Dodge *et al.*, 2011, 2014; Harris *et al.*, 2011). While the great majority of direct capture events have had no apparent negative impact on individual turtles, at least one unexplained mortality has occurred (personal communication from Scott Benson, Southwest Fisheries Science Center, NOAA Fisheries Service), and assessment of direct capture safety for this species remains an ongoing concern for researchers and regulatory agencies. For example, in the USA, federal authorities require veterinary personnel and cardiopulmonary resuscitation equipment to be present during leatherback direct capture events (e.g. NMFS ESA Permit 15672).

Due to the fact that these rare, large-bodied, pelagic turtles are difficult to locate, capture and handle, data on physiological effects of capture are very limited (Innis *et al.*, 2010; Harris *et al.*, 2011). In one previous study that assessed leatherback physiological status at a single time point during capture events, the turtles demonstrated a mild respiratory and metabolic acidosis in comparison to previously published data for unrestrained nesting females, similar to that seen in post-nesting female leatherbacks under the influence of anaesthesia (Harms *et al.*, 2007; Innis *et al.*, 2010). It is possible that this physiological state was a consequence of the capture event itself or may have been pre-existing due to the natural activity of the turtle immediately prior to capture (e.g. resting at the surface while recovering from a dive), or a combination of the two. To our knowledge, no study has attempted serial sampling of leatherbacks captured at sea to investigate the physiological effects of capture over time. In order to characterize better the physiological changes during direct capture of leatherback turtles, this study evaluated the cardiorespiratory and metabolic status of turtles immediately after capture and immediately prior to release. Our goals were as follows: (i) to determine whether captured turtles appeared physiologically normal upon release (comparing status upon release with the limited existing data for presumed healthy leatherback turtles); and (ii) to determine whether turtles exhibited any measurable changes in physiological status during the period of capture (comparing status immediately after capture with status immediately prior to release).

## Materials and methods

Leatherback turtles were captured off the coast of Massachusetts in August 2012, in order to investigate movement patterns, feeding ecology, habitat use and health status, under authorization of the United States Department of Commerce National Marine Fisheries Service (NMFS ESA Permit 15672). Veterinary personnel were deployed on each capture expedition as described by the National Marine Fisheries Service Procedures for Handling and Monitoring Leatherbacks During Capture-Related Work (NMFS ESA Permit 15672). Details of the capture, satellite tag attachment and health assessment methodologies have been described previously (Innis *et al.*, 2010; Dodge *et al.*, 2014). Briefly, turtles were spotted at sea by observers in an airplane or boat. Upon locating a turtle at the surface, a break-away hoop net with a purse-string closure mechanism was used to capture the turtle from the bow of the boat. The turtle then was secured on a ramp deployed from the stern and was brought onto the deck for evaluation, satellite tag application and diagnostic sample collection. Sea surface temperature (SST), time of capture and time of release were recorded. The duration of each handling event was defined as the time between net capture and release.

Once the turtle was secured onboard the vessel, venipuncture sites were disinfected by using sterile povidone iodine and isopropyl alcohol-infused gauze pads, and a 12–20 ml blood sample (hereafter termed the ‘post-capture sample’) was collected from the dorsal cervical sinus or dorsal caudal (tail) vein by using a 3.75–7.5 cm, 18- to 21-gauge needle attached to a heparinized syringe. Syringes were prepared by using liquid sodium heparin (heparin sodium, 1000 USP/ml; APP Pharmaceuticals, LLC, Schaumburg, IL, USA), which was repeatedly expelled from the syringe until no visible heparin remained, resulting in a heparin concentration of <10 USP/ml of blood. The time of blood collection relative to the time of capture was recorded.

A physical examination was conducted by the veterinary team while the satellite tag team attached the tag. Each turtle was measured (curved carapace length [CCL], measured from the nuchal notch to the pygal tip alongside the mid-line vertebral carapace ridge; and curved carapace maximal width [CCW]; Bolten, 1999), photographed and checked for external tags and internal passive integrated transponder (PIT) tags (Balazs, 1999). A deck hose was used to wet the turtle with ambient seawater, and a moist cloth was placed over the eyes to decrease visual stimuli. A flexible digital temperature probe was inserted ∼30 cm into the cloaca to record body temperature. The respiratory rate was recorded by visual monitoring. Determination of heart rate was attempted by using a Doppler blood flow detector (Pocket-Dop3; Nicolet Vascular, Madison, WI, USA) positioned dorsal to the hip as previously described (Innis *et al.*, 2010). Sex was assigned based on sexual dimorphism of the tail for turtles longer than 145 cm CCL (James *et al.*, 2007). For turtles with a CCL of <145 cm, sex was identified based on display of the penis during examination, or they were classified as unknown sex.

Before release, all the turtles that did not have pre-existing identification tags were marked with a single PIT tag in the dorsal shoulder musculature with a sterile syringe implanter (TX1440L 125 kHz tags; Biomark, Inc., Boise, ID, USA), and Inconel flipper tags (model 681; National Band and Tag Co., Newport, KY, USA) were applied to the thin fold of skin between the tail and the rear flippers (Balazs, 1999). Two skin samples were collected with sterile, disposable skin biopsy punches (4 mm Acu-Punch; Acuderm Inc., Fort Lauderdale, FL, USA) from the trailing edge of the rear flipper (Dutton, 1996). All tagging and biopsy sites were cleaned and disinfected with sterile povidone iodine and isopropyl alcohol-infused gauze pads before tag application or skin sampling. Upon completion of other procedures, immediately prior to release, a second, smaller volume of blood (∼2 ml; hereafter termed the ‘pre-release sample’) was collected with a 3 ml heparinized syringe as described above, and the time of blood collection relative to capture was noted. The pre-release sample was collected from the same general anatomical location as the post-capture sample (i.e. dorsal cervical sinus vs. tail), except that the contralateral side was used for collection of pre-release dorsal cervical sinus samples. Respiratory rate, heart rate and body temperature were assessed again, and the turtle was released.

After collection, blood-filled syringes were capped and placed in a cooler with ice until processed. As soon as possible (median 6.5 min, range 1–35 min; Table 2), whole blood samples were analysed directly from the collection syringes for pH, partial pressure of carbon dioxide (pCO_2_), partial pressure of oxygen (pO_2_) and concentrations of glucose, sodium, potassium, total carbon dioxide, ionized calcium and lactate, by using a point-of-care analyser (iSTAT with CG4+ and CG8+ cartridges; Abaxis, Union City, CA, USA) following the manufacturer's instructions. The remaining post-capture blood sample was then transferred to lithium heparin blood collection tubes (BD Vacutainer; Becton Dickinson) and placed on ice for later haematological and biochemical studies. A portion of the post-capture blood sample was centrifuged as soon as possible after collection (median 16 min, range 11–49 min) at 1500***g*** for 5 min, and the plasma was harvested and placed on ice for later biochemical studies. Upon return to shore, haematological and plasma biochemical samples were transferred on ice to a veterinary diagnostic laboratory (Idexx, North Grafton, MA, USA), refrigerated, and analysed within 24 h of collection as previously described (Innis *et al.*, 2010). Plasma from the post-capture blood sample was also frozen at −80°C prior to transfer to a veterinary diagnostic laboratory (Diagnostic Center for Population and Animal Health, Michigan State University, Lansing, MI, USA) for determination of plasma β-hydroxybutyrate concentrations as previously described (Innis *et al.*, 2010).

Results for blood pH, pCO_2_ and pO_2_ were mathematically corrected for each turtle's body temperature (pH_TC_, pCO_2TC_ and pO_2TC_), and pH-corrected ionized calcium values (iCa_cor_) were calculated by using pH_TC_ (Chittick *et al.*, 2002; Innis *et al.*, 2007). Values for αCO_2_ and the dissociation constant p*K* were calculated (Stabenau and Heming, 1993) and were used to calculate a HCO_3_^−^ value by using the Henderson–Hasselbalch equation, pH_TC_ and pCO_2TC_.

Statistical comparison of post-capture and pre-release data for each blood parameter was performed using Student's paired *t*-tests after confirming that data were normally distributed (InStat 3.0b for Macintosh OSX; GraphPad Software Inc., San Diego, CA, USA). Due to small sample sizes and multiple comparisons, α values were initially set at 0.05 and then adjusted for multiple comparisons using the Holm–Bonferroni sequential correction method (Holm, 1979; Abdi, 2010).

## Results

Seven turtles were captured during 3 days at sea between 2 and 9 August 2012. Direct capture events in this study proceeded smoothly and safely, with an average total event duration of 59 min and a maximal event duration of 67 min (Table 1; Supplementary Table 1). Turtles were judged to be in good health based on physical examination and clinical pathological data (Tables 1–3; Supplementary Tables 1 and 2). Six turtles had mild focal zones of dermatitis or dermal abrasion, while five turtles had previously healed, non-significant injuries of limb margins, as commonly seen in this species (Innis *et al.*, 2010; Harris *et al.*, 2011). Two turtles sustained minor injuries during the capture event due to tension of the capture net over skin surfaces, including perinasal skin abrasion (*n* = 1) and unilateral partial avulsion of the keratin tip of the upper rhamphotheca (*n* = 1). Three adult female turtles were found to have pre-existing PIT tags and flipper tags that had been applied during previous nesting events in French Guiana, Trinidad and St Kitts. Post-release satellite telemetry data will be documented elsewhere, but in summary, data indicated that turtles remained in good health after release, including a mean duration of satellite tag transmission time of 260 days (range 142–342 days), and long-distance migration to nesting grounds and winter foraging habitats (mean distance travelled 8638 km [range 3772–13 936 km]).

**Table 1:** Physical examination data, water temperature and temporal data recorded during direct capture and handling of seven leatherback turtles

Parameter	Mean	Median	SD	Minimum	Maximum
CCL (cm)	149	152	7.0	137	156
CCW (cm)	110	112	5.9	101	118
Initial body temperature (°C)	28.0	27.9	1.4	25.8	30.1
SST (°C)	21.3	20.5	1.6	20.2	24.2
Difference in body temperature/SST (°C)	6.7	6.6	0.9	5.5	8
Second body temperature (°C)	27.7	27.6	1.5	25.5	29.6
Time of second temperature (min post-capture)	55	58	6.9	45	60
Initial RR (breaths/min)	5	4	1.9	4	9
Second RR (breaths/min)	4	4	1.4	2	6
Time of second RR (min post-capture)	55	58	6.9	45	60
Initial HR (beats/min)	30	30	5.5	24	36
Second HR (beats/min)	33	34	3.8	28	36
Time of second HR (min post-capture)	57	60	6.5	47	60
Venipuncture time 1 (min post-capture)	26	25	7.2	15	37
Venipuncture time 2 (min post-capture)	51	53	9.3	39	60
Time between venipunctures (min)	25	27	6.5	17	35
Blood analysis time 1 (min post-collection)	12	4	15.3	1	35
Blood analysis time 2 (min post-collection)	10	8	6.2	2	20
Duration of event (min)	59	60	7.4	48	67

Abbreviations: CCL, curved carapace length; CCW, curved carapace width; HR, heart rate; RR, respiratory rate; SST, sea surface temperature.

**Table 2:** Paired post-capture (1) and pre-release (2) blood biochemical data recorded by a point-of-care analyser during direct capture and handling of seven leatherback turtles

Parameter	Turtle identity							Mean	Median	SD	Minimum	Maximum
	1	2	3	4	5	6	7					
Glucose 1 (mg/dl)	56	68	73	69	87	64	65	69	68	9.6	56	87
Glucose 2 (mg/dl)	60	60	72	69	88	62	54	66	62	11.3	54	88
Sodium 1 (mmol/l)	161	E	150	147	150	164	144	153	150	8.0	144	164
Sodium 2 (mmol/l)	160	151	144	143	150	163	144	151	150	8.0	143	163
Potassium 1 (mmol/l)^a^	3.2	E	5.5	4.4	3.5	3.7	5.1	4.2	4.1	0.9	3.2	5.5
Potassium 2 (mmol/l)	3.4	6.6	6.8	8.5	5.3	4.6	5.8	5.9	5.8	1.7	3.4	8.5
Lactate 1 (mmol/l)	10.4	2.1	10.6	3.1	7.4	12.3	8.2	7.7	8.2	3.9	2.1	12.3
Lactate 2 (mmol/l)	4.5	3.4	12.9	7.7	12.0	10.4	8.0	8.4	8.0	3.6	3.4	12.9
pH_TC_ 1^a,b^	7.44	7.26	7.23	7.25	7.29	7.25	7.33	7.29	7.26	0.07	7.23	7.44
pH_TC_ 2	7.47	7.40	7.32	7.36	7.35	7.33	7.49	7.39	7.36	0.07	7.32	7.49
pCO_2TC_ 1 (torr)	53.4	59.0	57.2	37.0	63.9	54.5	41.6	52.4	54.5	9.7	37.0	63.9
pCO_2TC_ 2 (torr)	46.2	31.3	33.2	52.6	42.9	48.4	31.6	40.9	42.9	8.8	31.3	52.6
pO_2TC_ 1 (torr)	84.9	55.6	66.8	40.4	56.9	71.5	63.8	62.8	63.8	14.0	40.4	84.9
pO_2TC_ 2 (torr)	85.8	87.0	89.9	51.8	69.2	60.9	66.4	73.0	69.2	14.7	51.8	89.9
HCO_3_^−^ 1 (mequiv/l)	39.6	27.9	25.1	18.0	34.1	26.0	24.9	28.0	26.0	7.0	18.0	39.6
HCO_3_^−^ 2 (mequiv/l)	36.2	30.2	26.2	45.5	36.5	39.6	37.4	36.0	36.5	6.3	26.2	45.5
iCa_cor_ 1 (mmol/l)	1.12	0.61	0.76	0.62	0.63	0.74	0.62	0.73	0.63	0.18	0.61	1.12
iCa_cor_ 2 (mmol/l)	0.93	0.46	0.62	0.57	0.75	0.64	0.58	0.65	0.62	0.15	0.46	0.93

Abbreviations: E, analyser error; iCa_cor_, pH-corrected ionized calcium; pCO_2_, partial pressure of carbon dioxide; pO_2_, partial pressure of oxygen; TC, temperature corrected.

^a^Significant difference between paired values, *P* < 0.05.

^b^Significant difference between paired values after Holm–Bonferroni correction.

**Table 3:** Post-capture haematological and plasma biochemical data for seven directly captured leatherback turtles

Parameter	Mean	Median	SD	Minimum	Maximum
Haematocrit (%)	41	43	5.1	34	47
White blood cells (cells/μl)	16 000	15 000	6240	9600	28 900
Heterophils (%)	50.7	51	4.6	44	57
Lymphocytes (%)	20.0	17	6.9	13	30
Monocytes (%)	2.6	3	1.0	1	4
Eosinophils (%)	26.4	29	6.3	16	34
Basophils (%)	0.3	0	0.5	0	1
Heterophils (cells/μl)	8012.7	8160	2859	5472	13 872
Lymphocytes (cells/μl)	3036.9	3400	968	1632	4060
Monocytes (cells/μl)	407.1	320	234	170	867
Eosinophils (cells/μl)	4488.3	4350	2692	1840	9826
Basophils (cells/μl)	55.0	0	109	0	289
ALP (U/l)	63.3	65	28	15	110
ALT (U/l)	14.3	13	6.0	7	25
AST (U/l)	141.7	139	57	42	224
CK (U/l)	265.3	64	446	36	1246
LDH (U/l)	418.9	349	257	186	947
Albumin (g/dl)	1.6	1.6	0.2	1.2	1.8
Total protein (g/dl)	4.4	4.6	0.6	3.4	4.9
Globulin (g/dl)	2.8	3	0.4	2.2	3.3
BUN (mg/dl)	131.0	125	15.3	113	154
Cholesterol (mg/dl)	284	273	154	57	540
Glucose (mg/dl)	76.3	76	10.6	63	95
Calcium (mg/dl)	6.2	6.2	0.4	5.7	6.9
Phosphorus (mg/dl)	7.3	5.9	3.5	3.2	13.2
Total CO_2_ (mequiv/l)	28.1	26	5.3	21	38
Chloride (mmol/l)	124.7	123	7.2	116	135
Potassium (mmol/l)	4.7	4.6	1.2	3.5	6.7
Sodium (mmol/l)	156.9	156	5.8	151	168
Uric acid (mg/dl)	1.3	1.5	0.4	0.4	1.7
Anion gap (mequiv/l)	9.0	10	5.2	2	15
Triglycerides (mg/dl)	765	926	326.5	160	1100
HDL (mg/dl)	38	41	19.1	11	70
LDL (mg/dl)	93	42	102.6	5	285
BHB (mg/dl)	1.2	1.1	0.6	0.5	2.4

Abbreviations: ALP, alkaline phosphatase; ALT, alanine aminotransferase; AST, aspartate aminotransferase; BHB, β-hydroxybutyrate; BUN, blood urea nitrogen; CK, creatine kinase; HDL, high-density lipoprotein; LDH, lactate dehydrogenase; LDL, low-density lipoprotein.

**Figure 1:**
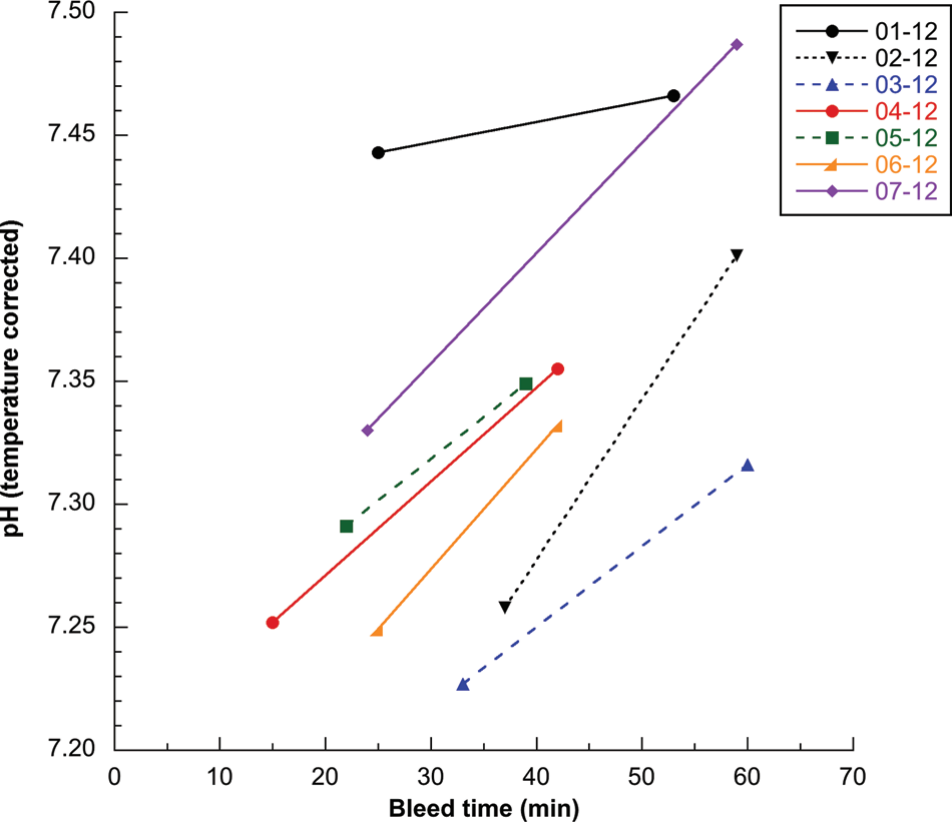
Post-capture and pre-release temperature-corrected blood pH vs. time for seven leatherback turtles. Turtle identification is indicated by number and year, correlated to turtles 1–7 as shown in Table 2.

**Figure 2:**
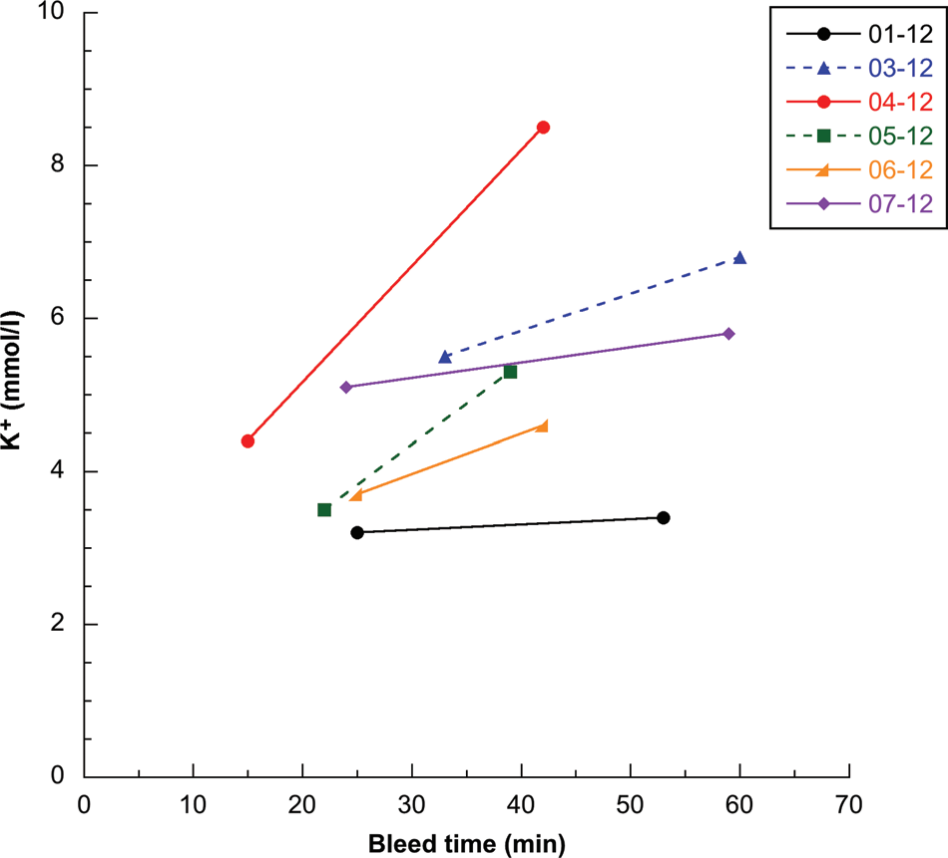
Post-capture and pre-release blood potassium concentrations vs. time for six leatherback turtles. Turtle identification is indicated by number and year, correlated to turtles 1–7 as shown in Table 2.

Body temperature, body temperature to sea surface temperature differential, respiratory rate, heart rate, haematological data and the majority of biochemical data were consistent with values previously reported for this species and were considered to be normal (Frair *et al.*, 1972; Lutcavage *et al.*, 1992; Paladino *et al.*, 1996; Southwood *et al.*, 1999; James and Mrosovsky, 2004; Deem *et al.*, 2006; Harms *et al.*, 2007; Myers and Hays, 2007; Innis *et al.*, 2010; Harris *et al.*, 2011; Honarvar *et al.*, 2011; Perrault *et al.*, 2012; Stewart *et al.*, 2012). Significant differences between paired post-capture and pre-release data were seen for only blood pH and potassium concentrations (*P* = 0.0018 and *P* = 0.0452, respectively; Table 2, Figs 1 and 2), with pH also being significantly different after Holm–Bonferroni correction. Consistent with limited data from a previous study (Innis *et al.*, 2010), turtles were initially affected by mild respiratory and metabolic acidosis (i.e. low venous pH and bicarbonate and high venous pCO_2_) in comparison to nesting females and some entangled leatherbacks. In comparison to initial post-capture status, pre-release data obtained on average 25 min later suggested that some degree of physiological recovery occurred during the on-board procedures, including a significant increase in pH, and trends toward decreasing pCO_2_, increasing pO_2_ and increasing bicarbonate. There was a significant increase in blood potassium concentrations between post-capture and pre-release blood samples, with post-capture results considered normal, and pre-release results indicating mild to moderate hyperkalaemia for several turtles compared with the majority of previous studies for this species (i.e. potassium > 6.5 mmol/l; Deem *et al.*, 2006; Harms *et al.*, 2007; Innis *et al.*, 2010; Harris *et al.*, 2011). Paired blood potassium data were available for only six turtles due to failure of the analyser to provide a post-capture result for one individual.

## Discussion

Here we provide the first serial physiological data obtained during leatherback turtle direct capture and handling events. The results of this study indicate that directly captured leatherback turtles were mildly acidotic initially, but that this status improved over time. We hypothesize that the improvement in respiratory status during the on-board events was the result of ongoing voluntary ventilation. Similar physiological recovery from submergence has been documented by serial sampling in other sea turtle species (Lutz and Bentley, 1985; Stabenau *et al.*, 1991; Harms *et al.*, 2003). This study does not answer the question of whether the initial acidosis observed in leatherbacks was caused by the capture event or whether it was pre-existing prior to capture; however, results do indicate that acidosis was not exacerbated during the time that on-board procedures were conducted. The observed respiratory rates (median 4 breaths/min, range 2–9 breaths/min) were similar to those previously observed for leatherback turtles out of water (Paladino *et al.*, 1996; Harms *et al.*, 2007; Innis *et al.*, 2010; Harris *et al.*, 2011), but could be considered hyperventilatory in comparison to the species typical dive intervals (e.g. typical dives are often 5 min in duration, and sometimes much longer; Southwood *et al.*, 1999; Dodge *et al.*, 2014). Hyperventilation could aid in recovery from acidosis. Respiratory and metabolic acidosis may occur in many species during conditions of physiological challenge, such as reduced cardiopulmonary function, various disease states and heavy exertion. In sea turtles, such derangements have been described as a result of extended submergence (natural and experimental), stranding, cold-stunning and general anaesthesia (Stabenau *et al.*, 1991; Chittick *et al.*, 2002; Harms *et al.*, 2003, 2007, 2014; Keller *et al.*, 2012; Camacho *et al.*, 2013). Acidosis has also been documented during a semi-natural 15 min dive of a tethered, catheterized green turtle (*Chelonia mydas*; Wood *et al.*, 1984). In future studies, differentiating whether such acidotic conditions arise during the normal dive repertoire of leatherbacks will probably require development of implanted blood sampling devices that can archive data for later retrieval or remote delivery (Cooke *et al.*, 2004).

Initial plasma potassium concentrations in captured turtles were within most previously published ranges for this species and were presumed to be normal (Deem *et al.*, 2006; Harms *et al.*, 2007; Innis *et al.*, 2010; Harris *et al.*, 2011; Honarvar *et al.*, 2011; Perrault *et al.*, 2012; Stewart *et al.*, 2012). Pre-release potassium concentrations were significantly higher than initial concentrations and higher than concentrations reported in most prior studies, but were similar to values documented by a direct capture study in the Pacific (Harris *et al.*, 2011). Increased serum potassium concentrations can be associated with exertional events in many species (Paterson *et al.,* 1989; Hanley *et al.*, 2005; Huerta-Alardín *et al.*, 2005). Exertional hyperkalaemia can be transient, with serum potassium returning to normal concentrations rapidly when exertion is stopped (Paterson *et al.*, 1989). However, in cases of severe exertional myopathy, serum potassium concentrations and serum muscle enzyme concentrations (e.g. creatine kinase, lactate dehydrogenase, aspartate aminotransferase) may increase over time due to rhabdomyolysis and subsequent acute renal failure (Hanley *et al.*, 2005; Huerta-Alardín *et al.*, 2005). While post-capture plasma muscle enzyme concentrations were considered normal for these turtles, this does not rule out myopathy, because it is known that enzyme concentrations may take hours or days to increase after muscle trauma (Hanley *et al.*, 2005; Huerta-Alardín *et al.*, 2005). The turtles' post-release behavioural data suggest that clinically relevant myopathy did not develop. Nonetheless, the consistent, significant increase in plasma potassium concentrations in captured turtles is notable and highlights the importance of physiological monitoring during capture events. These results are also likely to be relevant to unintentional leatherback capture events (e.g. fisheries interactions) when interactions may be more prolonged or extreme. It is possible that prolonged or particularly difficult capture events could lead to more severe hyperkalaemia and associated adverse cardiac effects. Cardiac monitoring of leatherback turtles during capture events has been accomplished by electrocardiography, Doppler blood flow detection and pulse oximetery (Harms *et al.*, 2007; Innis *et al.*, 2010; Harris *et al.*, 2011), and it is recommended that such modalities continue to be used because they could be helpful in detecting cardiac arrhythmias associated with hyperkalaemia.

The methodologies, limitations and general results of at-sea health assessment of leatherback turtles have been described in detail in previous reports (Innis *et al.*, 2010; Harris *et al.*, 2011). Relevant methodological data and general health data are provided here as context for paired physiological data, but detailed discussion of these points is beyond the scope of this report. In assessing physiological data, it is necessary to compare results with baseline values that are considered ‘normal’ for a given species. For leatherback turtles, blood analysis data have been obtained from nesting females, turtles entangled in fishing gear, anaesthetized post-nesting females, sedated and anaesthetized hatchlings and directly captured turtles (Deem *et al.*, 2006; Harms *et al.*, 2007, 2014; Innis *et al.*, 2010; Harris *et al.*, 2011; Honarvar *et al.*, 2011; Perrault *et al.*, 2012; Stewart *et al.*, 2012). None of these conditions necessarily represent ‘normal’ physiological conditions, but they provide the only available comparisons until blood data are available for unrestrained free-swimming wild leatherbacks.

While limited in sample size, this study provides further support for the general safety of leatherback direct capture events and provides further evidence that on-board physiological monitoring is valid and important for assessing the safety of such events. In general, the physiological status of turtles remained stable during capture and handling; however, the increase in blood potassium concentrations is of potential concern. As researchers continue to study the conservation status and ecology of this endangered species, consideration should be given to serial physiological monitoring during handling events such that morbidity and mortality may be avoided. Evaluation of the blood potassium status of a larger number of leatherback turtles during capture and handling events is warranted. With additional study, it is possible that methods may be established to minimize physiological changes during such events.

## Supplementary material

Supplementary material is available at *Conservation Physiology* online.

## Funding

This work was supported by the United States Department of Commerce, National Oceanic and Atmospheric Administration (grant number NA10NMF4720028 to the Massachusetts Division of Marine Fisheries through the Endangered Species Act Section 6 Program [www.nmfs.noaa.gov/pr/conservation/states/]).

## Supplementary Material

Supplementary Data
