# Supplementary material for: Serial assessment of the physiological status of leatherback turtles (Dermochelys coriacea) during direct capture events in the northwestern Atlantic Ocean: comparison of post-capture and pre-release data
Source: Conserv Physiol. 2014 Oct 30;2(1):cou048. doi: 10.1093/conphys/cou048 (PMC4806728; doi:10.1093/conphys/cou048)
Supplement: Supplementary Data [file supp_2_1_cou048__index.html]

Supplementary Data 

# Serial assessment of the physiological status of leatherback turtles (*Dermochelys coriacea*) during direct capture events in the northwestern Atlantic Ocean: comparison of post-capture and pre-release data

## Supplementary Data

Supplementary Data

**Files in this Data Supplement:**

- Supplementary Table 1 - docx file
- Supplementary Table 2 - docx file
