# Supplementary material for: Serial assessment of the physiological status of leatherback turtles (Dermochelys coriacea) during direct capture events in the northwestern Atlantic Ocean: comparison of post-capture and pre-release data
Source: Conserv Physiol. 2014 Oct 30;2(1):cou048. doi: 10.1093/conphys/cou048 (PMC4806728; doi:10.1093/conphys/cou048)
Supplement: Supplementary Data [file supp_cou048_cou048supp_table1.docx]

| turtle ID | 1 | 2 | 3 | 4 | 5 | 6 | 7 |
| --- | --- | --- | --- | --- | --- | --- | --- |
| Sex | U | F | F | M | F | M | F |
| CCL (cm) | 137 | 148 | 156 | 152 | 156 | 144 | 153 |
| CCW (cm) | 101 | 113 | 118 | 114 | 112 | 104 | 108 |
| Initial body temp (°C) | 29.4 | 30.1 | 27.3 | 27.3 | 28.2 | 25.8 | 27.9 |
| SST (°C) | 23.2 | 24.2 | 20.3 | 20.7 | 20.2 | 20.3 | 20.5 |
| delta body temp/SST (°C) | 6.2 | 5.9 | 7 | 6.6 | 8 | 5.5 | 7.4 |
| 2nd body temp (°C) | NR | 29.6 | 29.0 | 27.2 | 27.1 | 25.5 | 27.9 |
| time of 2nd temp (mins post-capture) | NR | 60 | 60 | 55 | 45 | 47 | 60 |
| initial RR (bpm) | 5 | 4 | 4 | 4 | 9 | 4 | 4 |
| 2nd RR (bpm) | NR | 3 | 6 | 4 | 4 | 2 | 3 |
| time of 2nd RR  (mins post-capture) | NR | 60 | 60 | 55 | 45 | 47 | 60 |
| initial HR (bpm) | 24 | 36 | 32 | 24 | ND | 28 | 36 |
| 2nd HR (bpm) | NR | 36 | 32 | NR | ND | 28 | 36 |
| time 2nd HR  (mins post-capture) | NR | 60 | 60 | NR | NR | 47 | 60 |
| Venipuncture site | T | T | T | T | J | J | J |
| Venipuncture time 1 (mins post-capture) | 25 | 37 | 33 | 15 | 22 | 25 | 24 |
| Venipuncture time 2 (mins post-capture) | 53 | 59 | 60 | 42 | 39 | 42 | 59 |
| Venipuncture time Δ (mins) | 28 | 22 | 27 | 27 | 17 | 17 | 35 |
| Blood analysis time 1 (mins post-collection) | 35 | 33 | 4 | 3 | 2 | 1 | 4 |
| Blood analysis time 2 (mins post-collection) | 16 | 20 | 7 | 2 | 12 | 8 | 6 |
| Duration of event (mins) | 55 | 67 | 64 | 60 | 48 | 52 | 66 |

Supplemental Table 1. Physical examination data, water temperature, and temporal data recorded during direct capture and handling of seven leatherback turtles. SD=standard deviation, CCL=curved carapace length, CCW=curved carapace width, temp=temperature, SST=sea surface temperature, RR=respiratory rate, HR=heart rate, NR=not recorded, ND=not detected, T=tail, J=jugular.
