# Supplementary material for: Serial assessment of the physiological status of leatherback turtles (Dermochelys coriacea) during direct capture events in the northwestern Atlantic Ocean: comparison of post-capture and pre-release data
Source: Conserv Physiol. 2014 Oct 30;2(1):cou048. doi: 10.1093/conphys/cou048 (PMC4806728; doi:10.1093/conphys/cou048)
Supplement: Supplementary Data [file supp_cou048_cou048supp_table2.docx]

| Turtle ID | 1 | 2 | 3 | 4 | 5 | 6 | 7 |
| --- | --- | --- | --- | --- | --- | --- | --- |
| Hematocrit (%) | 43 | 45 | 38 | 37 | 46 | 34 | 47 |
| White blood cells (cells/µL) | 17000 | 14000 | 28900 | 9600 | 16000 | 15000 | 11500 |
| Heterophil % | 48 | 44 | 48 | 57 | 51 | 56 | 51 |
| Lymphocyte % | 20 | 29 | 14 | 17 | 17 | 13 | 30 |
| Monocyte % | 1 | 4 | 3 | 3 | 2 | 2 | 3 |
| Eosinophil % | 31 | 23 | 34 | 22 | 30 | 29 | 16 |
| Basophil % | 0 | 0 | 1 | 1 | 0 | 0 | 0 |
| Heterophils (cells/µL) | 8160 | 6160 | 13872 | 5472 | 8160 | 8400 | 5865 |
| Lymphocytes (cells/µL) | 3400 | 4060 | 4046 | 1632 | 2720 | 1950 | 3450 |
| Monocytes (cells/µL) | 170 | 560 | 867 | 288 | 320 | 300 | 345 |
| Eosinophils  (cells/µL) | 5270 | 3220 | 9826 | 2112 | 4800 | 4350 | 1840 |
| Basophils (cells/µL) | 0 | 0 | 289 | 96 | 0 | 0 | 0 |
| ALP(U/L) | 15 | 66 | 110 | 60 | 65 | 59 | 68 |
| ALT(U/L) | 10 | 12 | 19 | 14 | 13 | 7 | 25 |
| AST(U/L) | 42 | 133 | 224 | 139 | 152 | 119 | 183 |
| CK(U/L) | 36 | 88 | 64 | 44 | 41 | 1246 | 338 |
| LDH(U/L) | 186 | 947 | 537 | 305 | 349 | 360 | 248 |
| Albumin  (g/dL) | 1.8 | 1.8 | 1.6 | 1.5 | 1.6 | 1.2 | 1.6 |
| Total protein (g/dL) | 4.8 | 4.8 | 4.9 | 3.9 | 4.6 | 3.4 | 4.3 |
| Globulin (g/dL) | 3 | 3 | 3.3 | 2.4 | 3 | 2.2 | 2.7 |
| BUN(mg/dL) | 125 | 113 | 143 | 154 | 122 | 142 | 118 |
| Cholesterol  (mg/dL) | 57 | 379 | 273 | 322 | 251 | 169 | 540 |
| Glucose  (mg/dL) | 63 | 78 | 76 | 83 | 95 | 72 | 67 |
| Calcium  (mg/dL) | 6.7 | 6.2 | 5.8 | 6.2 | 6.9 | 5.7 | 6.2 |
| Phosphorus  (mg/dL) | 3.2 | 9.5 | 9.4 | 5.9 | 13.2 | 5.7 | 4.3 |
| Total CO2  (mEq/L) | 38 | 26 | 21 | 31 | 29 | 26 | 26 |
| Chloride  (mmol/L) | 124 | 135 | 123 | 121 | 116 | 134 | 120 |
| Potassium  (mmol/L) | 3.5 | 5.6 | 6.7 | 4.6 | 3.7 | 3.8 | 5.2 |
| Sodium  (mmol/L) | 160 | 157 | 152 | 156 | 151 | 168 | 154 |
| Uric acid (mg/dL) | 0.40 | 1.50 | 1.30 | 1.20 | 1.6 | 1.7 | 1.6 |
| Anion gap (meq/L) | 2 | 2 | 15 | 9 | 10 | 12 | 13 |
| Triglycerides (mg/dL) | 160 | 1100 | 952 | 548 | 982 | 686 | 926 |
| HDL (mg/dL) | 11 | 47 | 41 | 46 | 25 | 27 | 70 |
| LDL (mg/dL) | 14 | 112 | 42 | 166 | 30 | 5 | 285 |
| BHB(mg/dL) | 0.54 | 1.87 | 0.88 | 1.20 | 0.8 | 1.08 | 2.37 |

Supplemental Table 2. Hematologic and plasma biochemical data for seven directly captured leatherback turtles. ALKP=alkaline phosphatase; ALT=alanine aminotransferase; AST=aspartate aminotransferase; CK=creatine kinase; LDH=lactate dehydrogenase; BUN=blood urea nitrogen; HDL=high density lipoprotein; LDL=low density lipoprotein, BHB=betahydroxybutyrate.
